# Optimal Chirality Enhances Long‐Range Fluctuation‐Induced Interactions in Active Fluids

**DOI:** 10.1002/advs.202509539

**Published:** 2025-09-03

**Authors:** Hashem Fatemi, Hamidreza Khalilian, Jalal Sarabadani, Reza Shaebani

**Affiliations:** ^1^ School of Quantum Physics and Matter Institute for Research in Fundamental Sciences (IPM) Tehran 19538‐33511 Iran; ^2^ Department of Theoretical Physics and Center for Biophysics Saarland University 66123 Saarbrücken Germany

**Keywords:** chiral active matter, collective behavior, fluctuation‐induced interactions, geometry‐induced optimization, structure formation

## Abstract

Understanding interactions between chiral active particles— self‐propelling and self‐rotating entities— is crucial for uncovering how chiral active matter self‐organizes into dynamic structures. Although fluctuation‐induced forces in nonequilibrium active systems can drive structure formation, the role of chirality remains largely unexplored. Effective fluctuation‐induced forces between intruders immersed in chiral active fluids are investigated and revealing that the impact of chirality depends sensitively on particle shape. For circular particles, increasing the self‐rotation to self‐propulsion ratio suppresses the interaction, reflecting a transition from rotating flocks to localized spinners. Contrarily, a striking collective behavior emerges for rodlike particles: vortices spontaneously form around the intruders, most pronounced at an optimal chiral angle where the mean curvature of particle trajectories matches the intruder boundary curvature, maximizing the effective force. The attractive and repulsive force regimes are mapped across chirality, propulsion, and intruder separation, offering new insights and principles for designing and controlling self‐assembled active systems.

## Graphical abstract

Chiral active particles move along curved trajectories arising from the interplay of propulsion and self‐rotation. How chirality influences fluctuation‐induced forces in active matter systems remains unclear. It is shown that particle shape dictates this effect: chirality suppresses forces for circular particles but can enhance them for elongated ones, where matched curvatures promote vortex formation, increasing collisions, and strengthening effective interactions.

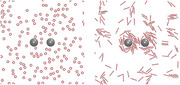

## 1 Introduction

Formation of patterns and structures is among the most remarkable features of active matter systems^[^ ^1^, ^2^, ^3^, ^4^ ^]^ Over the past two decades, significant progress has been made in understanding phenomena such as segregation, self‐assembly, and clustering in active‐passive mixtures, ^[^ ^5^, ^6^, ^7^ ^]^ motility‐induced phase separation, ^[^ ^1^ ^]^ and the emergence of effective interactions in confined active fluids^[^ ^8^, ^9^ ^]^ and actomyosin gels.^[^ ^10^, ^11^ ^]^ Most studies have focused on linear (i.e., non‐chiral) active systems, where rotational symmetry is preserved as particles reorient primarily through collisions or noise. In this class of active matter, the physics is governed by the interplay of propulsion, fluctuations, and nonlinearity and multiscale nature of interparticle interactions.

Nevertheless, lack of mirror symmetry is ubiquitous in both living and synthetic active systems. Examples include the clockwise^[^ ^12^, ^13^, ^14^, ^15^ ^]^ or counterclockwise^[^ ^16^ ^]^ circular motion of flagellated bacteria near surfaces, helical swimming of marine zooplankton ^[^ ^17^ ^]^ and sperm cells,^[^ ^18^, ^19^ ^]^ and chiral trajectories of synthetic self‐propelled objects with asymmetric shape^[^ ^20^, ^21^ ^]^ or mass distribution^[^ ^22^ ^]^ with respect to their propulsion axis. The coupling of translational and rotational modes of active motion is known to optimize navigation^[^ ^23^, ^24^, ^25^ ^]^ and induce gearlike rolling in dense confined geometries,^[^ ^26^, ^27^ ^]^ suggesting that the coupling may also affect fluctuation‐induced forces generic to nonequilibrium systems.^[^ ^28^, ^29^ ^]^ To get insight into how chiral active matter organizes into dynamic patterns and structures,^[^ ^30^, ^31^ ^]^ a detailed understanding of the interplay between active rotation and other key aspects of the problem is crucial, which yet remains elusive.

Fluctuation‐induced (FI) forces act across different length scales in nonequilibrium systems of driven passive^[^ ^32^, ^33^, ^34^ ^]^ or active ^[^ ^35^, ^36^, ^37^, ^38^, ^39^, ^40^, ^41^, ^42^, ^43^ ^]^ particles. While FI forces are typically weak in passive systems, they can become significantly enhanced with increasing activity.^[^ ^35^ ^]^ The force strength depends sensitively on bath properties such as density and noise strength, as well as on the size, shape, orientation, and separation of intruder particles. ^[^ ^32^, ^34^, ^35^, ^36^, ^37^, ^41^, ^42^ ^]^ Moreover, switching between attractive and repulsive forces have been observed upon varying the bath density or separation and size of the intruders.^[^ ^32^, ^35^, ^36^, ^37^, ^38^ ^]^ Whether and how the interplay of FI forces and other interactions can prevent or promote formation of structures in chiral active matter systems has remained unexplored to date. The subject is of fundamental and practical importance as, for example, in biofilm formation and design of micro‐devices^[^ ^44^ ^]^ and active chiral crystals. ^[^ ^45^, ^46^, ^47^ ^]^ A major step forward is to clarify how chirality influences FI interactions— a question only recently touched upon,^[^ ^38^, ^48^ ^]^ where relatively short‐range FI forces were found to arise from the chirality‐dependent spatial distribution of active bath particles.

**Figure 1:**
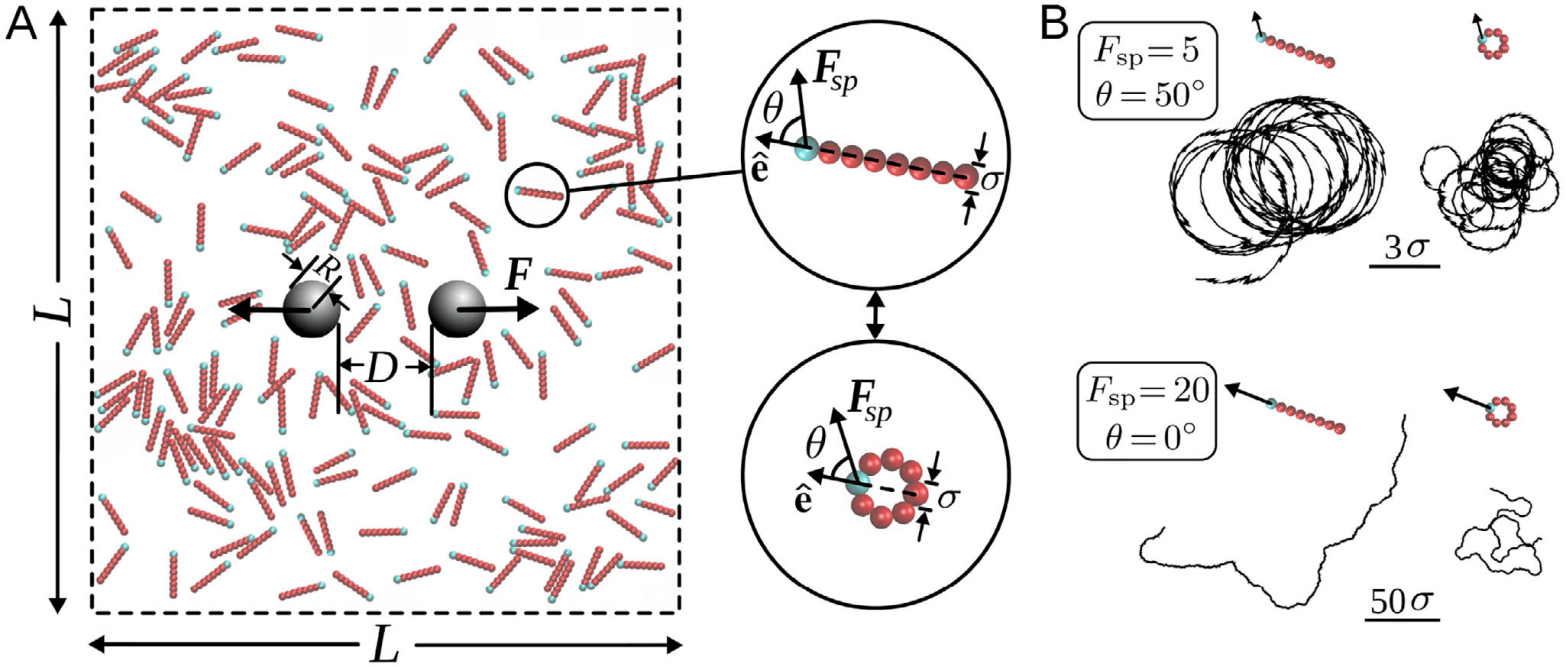
Schematic of the simulation box. A) Typical snapshot of the system with area fraction ϕ  =  0.1. Immobile (gray) intruders are immersed in an active bath consisting of either rodlike or circular rigid composites of beads as displayed in the insets. The self‐propulsion (active) force $F_{\text{sp}}$ is exerted on the head (blue) bead with the self‐propulsion angle (chirality) θ with respect to the intrinsic orientation $\hat{e}$ of the active object. B) Sample trajectories of active objects during the same time interval for different choices of chirality θ and magnitude of active force $F_{\text{sp}}$ (in 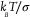 units).

Here, we address this open problem by investigating the effective interactions between immobile intruders immersed in chiral active fluids (**Figure** **1**A) using extensive Langevin dynamics simulations. We explore how the combination of chirality and elongation shapes the effective long‐range FI forces beyond the depletion interaction range. Specifically, we demonstrate that particle elongation plays a pivotal role: in dense environments, pure rotation leads to localized spinning for circular particles, while promoting correlated rotations for rods. To disentangle the effects of shape and chirality, we compare two limits— circular and rodlike active particles— and show that rods can generate significantly stronger FI forces under identical conditions. Remarkably, we uncover a nonmonotonic dependence of the FI force on chirality in rodlike active baths, revealing an optimal chiral angle that maximizes interaction strength and range. We trace this optimum to a spontaneous collective behavior in rodlike active baths: vortex formation and disruption around the intruders. The interplay between the mean curvature of active particle trajectories and the boundary curvature of the intruders governs the strength of this phenomenon. When these curvatures match, the strongest and most persistent vortices and thus the highest particle‐intruder collision rates occur, which enhance the FI interaction. We further establish a phase diagram mapping attractive and repulsive force regimes as functions of chirality, propulsion strength, and intruder separation.

## 2 Chiral Active Fluids

We construct rigid composites of touching beads of diameter σ arranged either on a straight line or on a circular ring; see Figure 1A. These composite objects constitute the elements of the bath. Here, we present results for eight‐bead composites. We consider a 2D system motivated by chiral in‐plane motion of biological and synthetic agents above surfaces.^[^ ^12^, ^13^, ^14^, ^15^, ^16^, ^20^, ^21^ ^]^ Periodic boundary conditions are imposed in both directions and two immobile circular intruders of radius *R*  =  5 σ are immersed with separation *D*. By exerting a self‐propulsion force $F_{\text{sp}}$ on each composite particle, the bath changes from a passive to an active fluid with a tunable forward propulsion. $F_{\text{sp}}$ is applied on the head bead of the rod or a chosen bead of the circular object (blue beads in Figure 1A). The chirality is tuned by changing the angle θ between $F_{\text{sp}}$ and the intrinsic orientation $\hat{e}$ of the active object (i.e., the line symmetrically dividing the composite through the blue bead). The choice of θ  =  0 (θ  ≠  0) corresponds to a non‐chiral (chiral) active fluid.

The intruders and the constituent elements of the active/passive bath are assumed to be rigid; thus, an exclusion interaction between particles is introduced through the Weeks‐Chandler‐Andersen potential^[^ ^49^ ^]^

(1) $$U_{\text{WCA}}\left(d\right)=\left\{\begin{array}{cc} U_{\text{LJ}}\left(d-\Delta \right)-U_{\text{LJ}}\left(d_{c}\right) & \text{if}\;d\;-\;\Delta \;<\;d_{c},\\ 0 & \text{if}\;d\;-\;\Delta \;\geq \;d_{c,} \end{array}\right.$$

with *d* being the center‐to‐center distance between the interacting objects, *U*_LJ_ the Lennard‐Johnes potential, and *d*_c_  =  2^1/6^σ the cut‐off distance. Δ equals 0 or *R*  −  σ/2 for bead‐bead (of different particles) or bead‐intruder interaction, respectively. The position 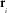 of the *i*th bead of each composite object evolves according to the Langevin equation

(2) $$M\;{\text{r̎}}_{i}=-\eta \;{\dot{r}}_{i}-\nabla U_{i}+\xi_{i}+F_{\text{sp},i}\;\delta_{\mathrm{hi}},$$

where *M*, η, and 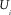 are the mass of each bead, solvent friction, and sum of all interactions acting on the *i*th bead, respectively. The random force 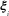 is a Gaussian white noise with zero mean and correlation 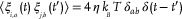, in which *a* and *b* denote Cartesian components of the vectors, *k*_B_ the Boltzmann constant, and *T* the temperature. The constraint that the active force only acts on the head (*h*) bead is enforced by the Kronecker delta in the last term. A few examples of the resulting particle trajectories at area fraction ϕ  =  0.1 are shown in Figure 1B. It is known that increasing self‐propulsion enhances forward motion and the asymptotic diffusion coefficient of non‐interacting active agents,^[^ ^50^, ^51^ ^]^ while inducing asymmetry in propulsion reduces the diffusion coefficient and leads to spiral trajectories.^[^ ^52^ ^]^ Similar trends are observed here upon varying the active force and chirality at low densities of the active bath. In the nonequilibrium steady state we measure the net force exerted by the active fluid on each intruder along the line which connects their centers. The force acting on the right intruder is denoted by F in the following. Because of large fluctuations, the force is measured for 10^4^ successive time intervals in the steady state and ensemble averaged over 10^2^ uncorrelated trajectories; see Experimental Section for more details.

## 3 Long‐Range Fluctuation‐Induced Interactions

**Figure 2:**
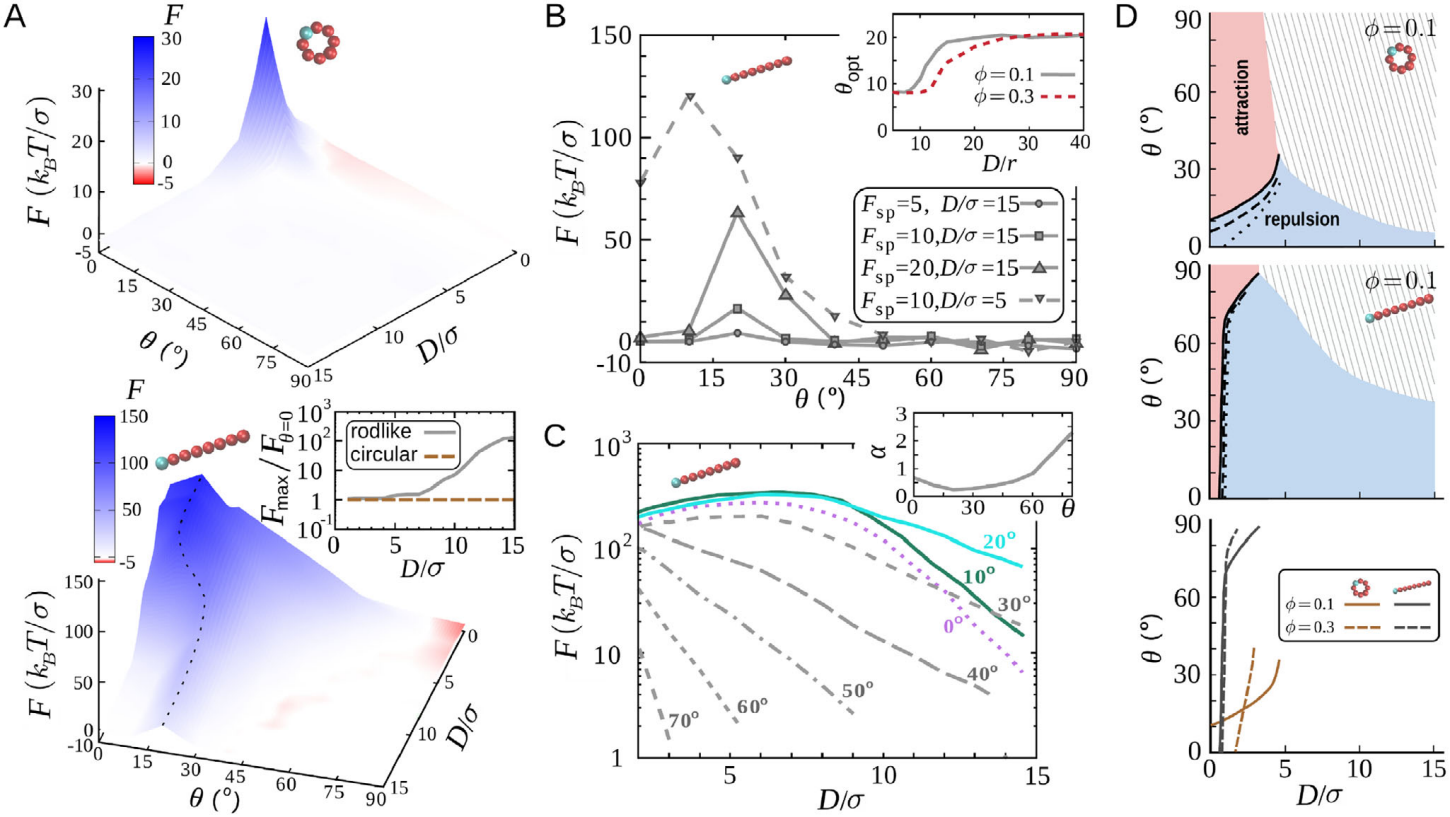
FI forces in chiral (θ≠0) active fluids. A) FI force *F* in terms of scaled gap size *D*/σ and chiral angle θ at $F_{\text{sp}}=10\;k_{B}T/\sigma$ and ϕ = 0.1 for circular (top) and rodlike (bottom) active objects. The dashed line in the lower panel indicates the location of the maximum force at different separations. The inset shows the ratio between the maximum FI force $F_{\max}$ and the non‐chiral FI force *F*_θ = 0_ at different values of *D*/σ for circular and rodlike objects. B) *F* versus θ in active baths of rodlike objects with ϕ = 0.1 for different values of $F_{\text{sp}}$ and *D*/σ. Inset: Optimal chiral angle $\theta_{\text{opt}}$ versus *D*/σ for different bath densities. C) *F* versus *D* for rodlike active particles at different values of chirality θ. Other parameters: $F_{\text{sp}}=10\;k_{B}T/\sigma$, ϕ = 0.1. Inset: Decay exponent α of the exponential fit to the *F*‐*D* tail behavior in terms of θ. D) Phase diagram of force sign switching in the (*D*, θ) plane for $F_{\text{sp}}=20$. The upper and middle panels represent the diagram for active chiral baths of circular or rodlike particles, respectively. The solid line marks the interface for $F_{\text{sp}}=20\;k_{B}T/\sigma$. The interface position is also shown for $F_{\text{sp}}=10$ (dashed line) and 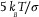 (dotted line). The hatched zones correspond to weak FI forces where random sign reversals occur within the accuracy of our measurements. The lower panel represents a comparison between the interface positions at bath densities ϕ = 0.1 and 0.3 for $F_{\text{sp}}=20\;k_{B}T/\sigma$.

To see how the FI force is influenced by chirality, we vary the chiral angle θ and measure *F* for different gap sizes *D* between the intruders. Interestingly, the results reveal that the impact of θ on *F* depends on the elongation of active particles. For circular particles, increasing θ monotonically weakens *F* (**Figure** **2**A). It means that for any given separation *D*, the intruders in an active fluid with circular constituent elements experience the maximum FI force in the non‐chiral case (θ  =  0). Contrarily, in case of rodlike active particles *F* behaves nonmonotonically upon increasing θ; it develops a peak at a nonzero chiral angle 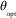 and decays at larger chiral angles. The inset of Figure 2A shows that the relative strengthening of *F* compared to the non‐chiral case is enhanced with increasing *D*. At large separations, where the force in the non‐chiral case is weak, adopting the optimal chiral angle can strengthen the force by several orders of magnitude.

In Figure 2B, we plot *F* versus θ for different values of $F_{\text{sp}}$ and *D* at bath density ϕ  =  0.1. It can be seen that the optimal chiral angle 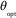 is independent of the choice of self‐propulsion force. In addition, 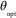 slightly grows with *D* at small separations but eventually saturates to 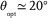 at larger *D* values (inset of Figure 2B). Similar conclusions are drawn for other values of bath density, though the transition to asymptotic 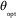 shifts to larger *D* values with increasing ϕ.

The optimal chirality also remarkably enhances the range of FI interactions. Figure 2C shows *F* in terms of *D* for different values of θ. The tail of the FI force versus *D* can be approximated by an exponential decay *F*_tail_  ∼  e^−α(θ) *D*/σ^ for each choice of the chiral angle. The decay slope α depends on the chirality: As shown in the inset of Figure 2C, by adopting a chiral angle around the optimal angle 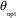, α reaches a minimum value corresponding to the longest range for the FI interaction. Moreover, Figure 2C shows a novel feature in active fluids: The FI force behaves nonmonotonically as a function of the separation between the intruders. It first develops a peak— at a value of *D* which can be beyond the short depletion range— and then decays. This feature appears regardless of active particle shape, though the peak is more pronounced for elongated particles.

Our results also reveal that in chiral active fluids, the sign of the FI force is determined in general by the interplay of chirality, self‐propulsion, bath density, and separation between the intruders. In Figure 2D, we present a cut through the phase diagram of FI force sign reversal, where the separation and chirality are changed. For both circular and rodlike active particles, increasing θ shifts the transition from attraction to repulsion to larger separations. $F_{\text{sp}}$ acts in the opposite direction, i.e., pushes the repulsive force zone to smaller *D* values (more pronounced for circular active particles); see top and middle panels of Figure 2D. The impact of bath density on the force sign depends on the chirality of the active particles: At small values of θ, increasing ϕ enhances the attractive force domain. The effect is more pronounced for circular active particles. In contrast, increasing ϕ at large chiral angles reduces the attraction domain to smaller separations (lower panel). We note that the hatched zones in the phase diagrams of Figure 2D mark the regions where the FI force is too weak (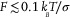) and random sign switchings occur within the accuracy of our time‐consuming measurements. Increasing $F_{\text{sp}}$ or ϕ strengthens the FI force and pushes the hatched zone to the right, i.e., larger separations.

## 4 Collision Statistics Around Intruders

To uncover the physical mechanisms driving fluctuation‐induced (FI) forces in chiral active systems, it is essential to analyze the distribution of thermodynamic fields around the intruders. In randomly driven passive baths, it has been shown that large intruder objects alter pressure field fluctuations in the confined region between them due to the boundary conditions they impose, resulting in effective long‐range interactions.^[^ ^32^, ^33^ ^]^ More recently, it was demonstrated that a probe placed near lateral walls in an active fluid experiences an effective force linked to the asymmetric distribution of active particles around it.^[^ ^8^ ^]^

**Figure 3:**
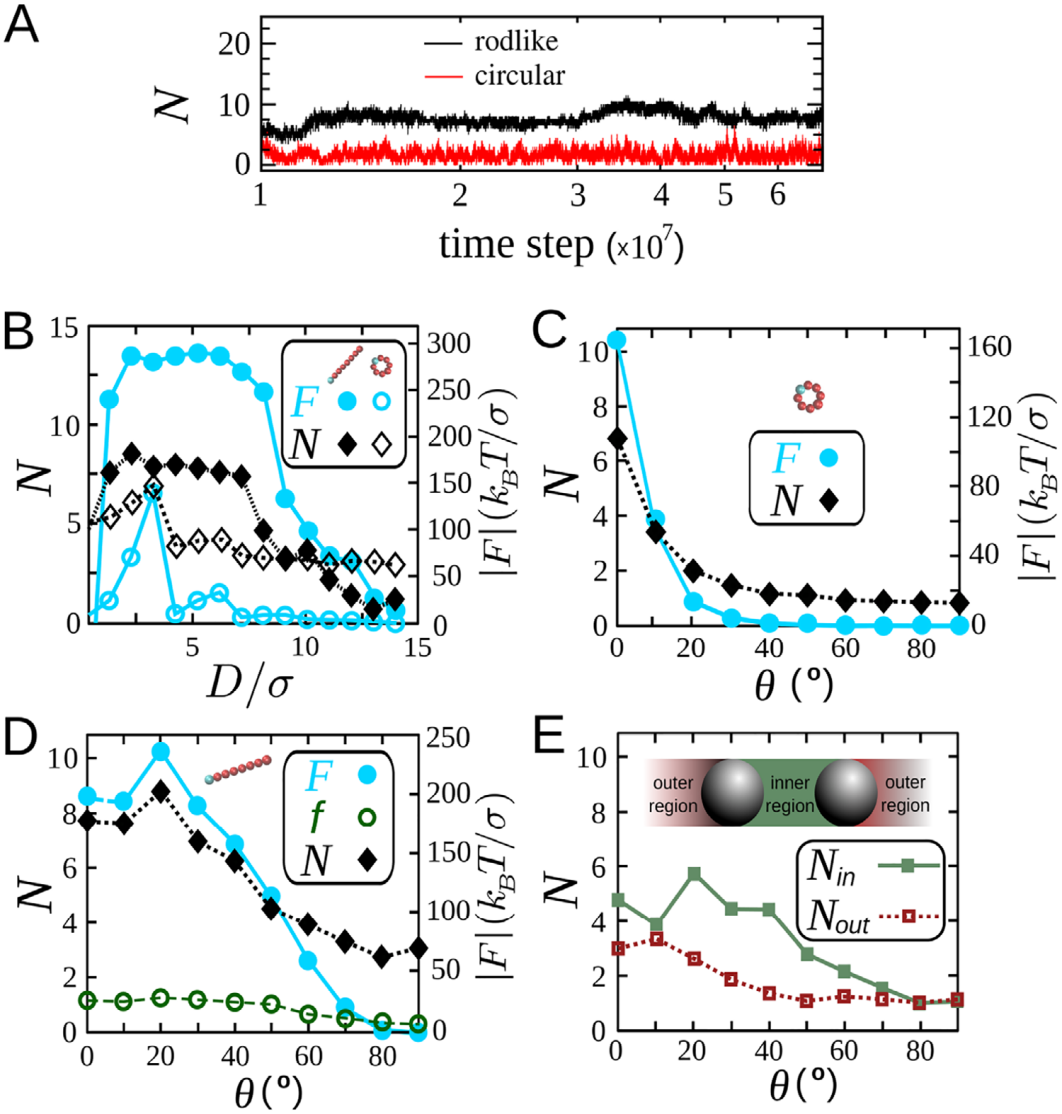
Statistics of collisions between active particles and intruders. A) Time evolution of the number of collisions (per 10^3^ time steps) between the right intruder and active particles in the steady state (time steps >10^7^), for ϕ  =  0.3, *D*/σ  =  8, $F_{\text{sp}}=\;20\;k_{B}T/\sigma$, and θ  =  20°. B) Mean number of collisions *N* with the right intruder and corresponding FI force *F* as functions of separation *D* for both rodlike and circular particles at $F_{\text{sp}}=\;20\;k_{B}T/\sigma$, ϕ  =  0.1, and θ  =  0. C,D) *N* and *F* versus chiral angle θ for $F_{\text{sp}}=\;20\;k_{B}T/\sigma$ in systems with circular particles (ϕ  =  0.3, *D*/σ  =  3) and rodlike particles (ϕ  =  0.1, *D*/σ  =  2), respectively. Panel (D) also shows the mean magnitude of force per collision, *f*, in terms of θ. E) Collision counts for rodlike particles in panel (D), separately shown for the inner and outer regions between intruders, as indicated in the inset.

We quantify the collision statistics between active particles and intruders, which serve as a direct measure of the asymmetry in thermodynamic fields and thus provide insights into the origins of FI forces. We hypothesize that the observed nonmonotonic dependence of the FI force on chirality or intruder separation stems from a similarly nonmonotonic dependence of the collision imbalance around the intruders. To test this hypothesis, we measure the steady‐state number of particle‐intruder collisions (**Figure** **3**A). Representative examples of the dependence of the force *F* and mean number of collisions *N* on separation *D* for both particle shapes are shown in Figure 3B, revealing a strong correlation between *F* and *N*. Likewise, the nonmonotonic variation of *F* with chirality θ in rodlike active fluids mirrors that of *N*. A comparison of circular and rodlike particle systems (Figures 3C,D) shows that while *N* decays monotonically with θ for circular particles, it exhibits a pronounced peak for rods— paralleling the behavior of *F*. This correlation establishes a direct link between *F* and *N*. Furthermore, by separating collisions in the inner and outer regions in Figure 3E, we confirm that the nonmonotonic behavior of *N* with θ arises from enhanced collisions in the inner region. We note that, in addition to the number of collisions, the mean force per collision also exhibits a slight peak at intermediate chiral angles (Figure 3D). Another point is that the sign reversal of the FI force toward attraction can occur when the collision rate in the inner region becomes lower than in the outer region. This can happen, for example, when particles trapped in the inner region collide with incoming particles from a denser surrounding bath and block their entry into the gap, thereby reducing the collision frequency in the inner region relative to the outer region.

## 5 Vortex Formation Around Intruders

**Figure 4:**
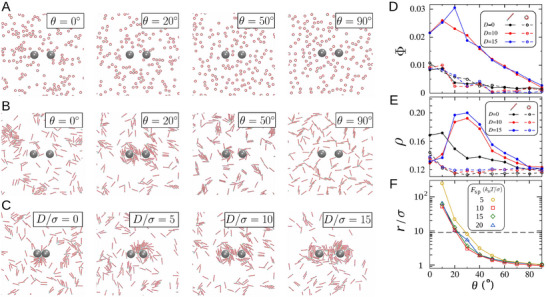
Vortex formation around intruders in chiral active baths of rodlike particles. A–C) Typical steady‐state configurations of chiral active particles around the intruders. The active bath has density ϕ  =  0.1, and particles are driven by an active force $F_{\text{sp}}=\;10\;k_{B}T/\sigma$. In panels (A) and (B), the intruder separation is *D*/σ  =  10, and circular or rodlike particles have chiral angles θ  =  0, 20, 50, or 90°. In panel (C), rodlike particles have θ  =  20°, and the intruder separation varies as *D*/σ  =  0, 5, 10, or 15. D) Vortex order parameter Φ as a function of chiral angle θ for circular and rodlike active particles at ϕ  =  0.1, $F_{\text{sp}}=\;10\;k_{B}T/\sigma$, and different intruder separations. E) Number density of chiral active bath particles around the intruders, ρ, as a function of θ, shown for the same set of conditions as in panel (D). F) Radius of curvature of the spiral trajectories (traced by the center of mass) of rodlike particles as a function of chiral angle, shown for different values of $F_{\text{sp}}$. The horizontal dashed line indicates the effective vortex radius near the intruders is obtained as the intruder radius plus half the rod length.

The emergence of a peak in particle‐intruder collisions at intermediate chiral angles θ  ≈  20° for elongated active particles raises the question of its underlying mechanism. To investigate this, we examine the dynamics of chiral active particles in the presence of intruders. As shown in **Figure** **4**A (see also Movie S1, Supporting Information), increasing θ for circular particles leads to a gradual transition from rotating flocks to localized spinners, largely independent of intruder presence. In contrast, for rodlike particles, a striking collective behavior arises: rods spontaneously organize into vortices around the intruders (Figure 4B; Movie S2, Supporting Information). We quantify this effect by computing the local mean velocity field v(r) around the intruders and define the vortex order parameter as $\Phi =\frac{\langle |v\cdot t|\rangle /\langle |v|\rangle -2/\pi }{1-2/\pi }$, where t is the unit vector tangent to the (clockwise) circular path around each intruder.^[^ ^53^ ^]^ Φ  =  1 corresponds to steady azimuthal circulation, while small values of Φ indicate a disordered chaotic flow. Vortex formation is highly dynamic: vortices around both intruders interfere with one another, leading to continual cycles of formation and disruption. This stochastic crowding around the intruders enhances particle‐intruder collisions. Notably, Φ peaks around θ  ≈  20° for rodlike active particles, coinciding with the highest collision rate and the strongest FI force (Figure 4D). For circular particles, by contrast, Φ remains weak and decreases with increasing θ. Figure 4E shows the number density of chiral active bath particles around the intruders (up to a distance *R* from the intruder surface), which exhibits a dependence on the chiral angle similar to that of the vortex order parameter.

The dependence of vortex formation on particle elongation arises from differences in the dynamics of individual circular and rodlike active particles. When the propulsion direction and intrinsic orientation of an active particle are misaligned, two key effects arise. First, forward propulsion is reduced, which shortens the directional correlation length;^[^ ^54^ ^]^ this effect impacts both circular and rodlike particles similarly. Second, the misalignment induces a torque that leads to self‐rotation and spiral trajectories, as illustrated in Figure 1B. Such torque can arise from viscous interactions, for example, between the cell body and flagella of swimming bacteria near surfaces, which alter local pressure and flow fields. The curvature of these trajectories depends on shape: for bacteria, the radius increases with cell‐body length,^[^ ^13^, ^16^ ^]^ and in asymmetric artificial microswimmers, the curvature is largely independent of propulsion strength but governed by shape asymmetry.^[^ ^20^ ^]^ The mean radius of the particle trajectory *r* can be roughly estimated by assuming that the translational and angular velocities are proportional to the forward propulsion force and the torque about the center of mass, respectively.^[^ ^55^ ^]^ Neglecting noise, this yields r∝cotθ, indicating that the spiral radius is a decreasing function of θ and is independent of the propulsion force. By extracting the mean radius of spiral trajectories from simulations, traced by the center of mass, we confirm that rodlike particles exhibit larger spiral radii than circular ones at the same chiral angle, leading to more correlated dynamics and stronger FI forces. As chirality increases toward θ  =  90°, where propulsion vanishes, rodlike particles continue to exhibit collective, entangled motion due to gearlike rolling. In contrast, circular particles gradually transition from vortex‐like patterns and rotating flocks to isolated spinners with no net displacement.^[^ ^45^, ^56^ ^]^ As shown in Figure 4F for rodlike particles, the trajectory radius decreases with increasing chirality. Crucially, around θ  ≈  20°, the trajectory radius closely matches the effective vortex radius near the intruders— defined as the intruder radius plus half the rod length. This geometric matching enhances vortex stability, maximizing particle‐intruder collisions and thus the FI force. It can also be seen that the θ‐dependence of the trajectory radius is largely unaffected by propulsion strength, which explains why the optimal chiral angle 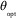 remains largely independent of $F_{\text{sp}}$.

## 6 Discussion and Conclusion

Having established that vortex formation and disruption underlie the enhanced particle‐intruder collisions and the chirality‐dependent optimization of FI forces, we now address the origin of the observed force optimization with respect to intruder separation. It is known that the mean time for an active searcher to encounter a target— or, equivalently, the cover time to scan a confined space— reaches a minimum at an optimal intermediate confinement size relative to the persistence length of the searcher.^[^ ^23^ ^]^ Analogously, for a given propulsion strength $F_{\text{sp}}$ in a non‐chiral active bath, we expect an optimal intruder separation *D* that minimizes the mean hitting time of intruders (targets) by active bath particles (searchers) in the inner confined region between the intruders (see inset of Figure 3E). This maximizes the collision frequency and thus the FI force. In contrast, changes in *D*, at given $F_{\text{sp}}$ and θ, have a smaller impact on collision rates in the outer region, where confinement varies less significantly.

In chiral baths of rodlike particles, the dependence of collision statistics on intruder separation *D* is strongly influenced by the ability of the system to form and sustain vortices. As shown in Figure 4C for chirality θ  =  20° (see also Movie S3, Supporting Information), vortices cannot develop at very small separations. A minimum separation— at least comparable to the length of the rodlike particles— is required to allow vortex formation around the intruders and enable their mutual interaction. This is also evident in Figure 4D, where the vortex order parameter remains weak for small *D*, and the optimal chiral angle for rods emerges only when the separation is sufficiently large. This observation further explains why the optimal chirality in the inset of Figure 2B reaches a plateau only at larger values of *D*. We also note that at small separations, the absence of vortex formation— which underlies the emergence of the optimal chiral angle and the associated force maximization— leads to a monotonically decreasing FI force with increasing θ. For rodlike baths at small *D*, this force is predominantly attractive.

**Figure 5:**
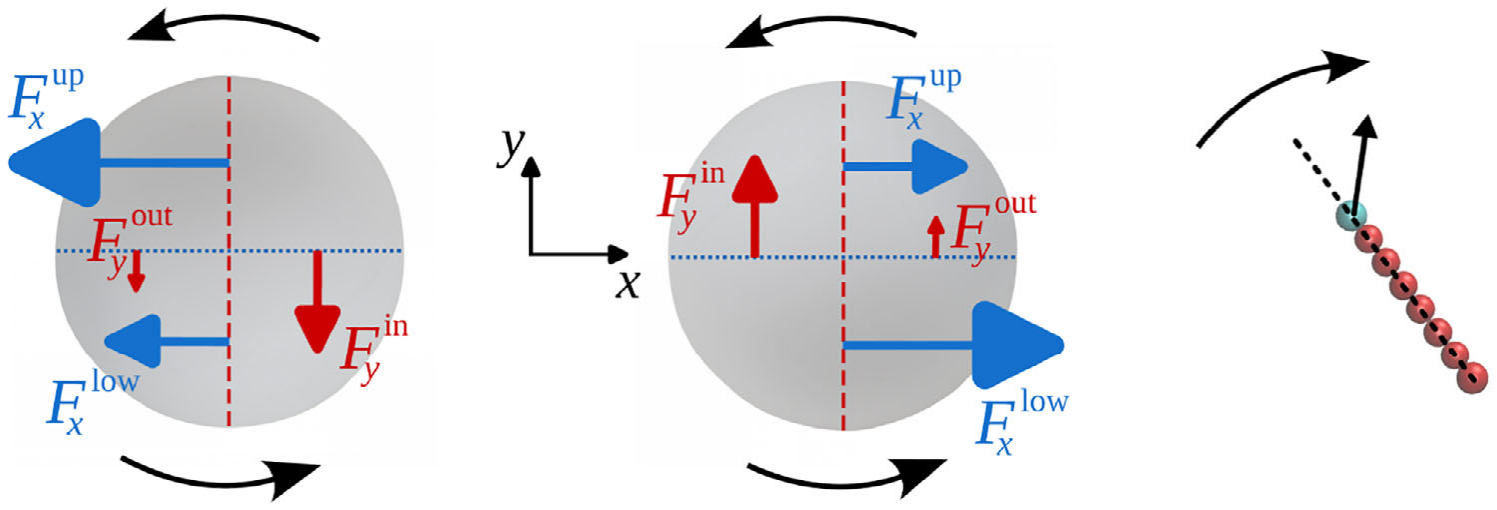
Schematic of the FI force components. The inner and outer (upper and lower) halves of each intruder are separated by the red dashed (blue dotted) lines. Examples of net force imbalances between different halves are: $\Delta F_{x}=\left|F_{x}^{\;\;\text{up}}-F_{x}^{\;\;\text{low}}\right|\;≃\;40.5$ vs $\Delta F_{y}=\left|F_{y}^{\;\;\text{in}}-F_{y}^{\;\;\text{out}}\right|\;≃\;27.7\;k_{B}T/\sigma$ for ϕ  =  0.1, and Δ*F*_*x*_  ≃  21.95 vs $\Delta F_{y}\;≃\;17.87\;k_{B}T/\sigma$ for ϕ  =  0.3. Other parameters: *D*/σ = 3, θ = 30°, and $F_{\text{sp}}=20k_{B}T/\sigma$. In these examples, the horizontal forces are repulsive, and the perpendicular force acting on the right (left) intruder is upward (downward). The black arrows around the intruders indicate their possible rotation direction when tangential contact forces are present.

Finally, we note that the unidirectional rotation of chiral active particles can exert a net torque on each intruder.^[^ ^57^ ^]^ By introducing tangential contact forces, particle‐intruder interactions can induce a gear‐like rotation— even in the case of a single intruder subject to isotropic collisions. We find a systematic imbalance in the horizontal force (along the *x*‐axis in **Figure** **5**) exerted on the upper versus lower halves of each intruder, quantified by $\Delta F_{x}=\left|F_{x}^{\;\;\text{up}}-F_{x}^{\;\;\text{low}}\right|\;>\;0$. Likewise, there is a net vertical force imbalance between the inner and outer halves of each intruder, $\Delta F_{y}=\left|F_{y}^{\;\;\text{in}}-F_{y}^{\;\;\text{out}}\right|\;>\;0$. In the presence of tangential contact forces, each intruder can experience a net torque due to the force imbalances Δ*F*_*x*_ and Δ*F*_*y*_ which act in opposite directions but do not cancel (Δ*F*_*x*_  >  Δ*F*_*y*_). Consequently, the intruders can rotate in the direction opposite to that of the active particles, as illustrated in Figure 5.

In summary, we have demonstrated that the interplay between propulsion, chirality, and particle shape dramatically modulates long‐range fluctuation‐induced forces between objects immersed in chiral active fluids. Specifically, we show that in systems of elongated active particles, the magnitude and range of the FI force can be significantly enhanced by tuning chirality, with an optimal chiral angle promoting vortex formation and maximizing interaction strength. Moreover, confinement between intruders introduces an optimal separation that further amplifies these interactions by increasing particle‐intruder encounter rates. These effective forces can play a critical role in driving self‐organization and assembly in active matter, particularly at microscales. Analogous phenomena have been observed in biological systems, where stress fluctuations in actomyosin gels induce attraction toward confining boundaries,^[^ ^10^ ^]^ and cytoskeletal dynamics drive nuclear fluctuations.^[^ ^11^ ^]^

Our findings position chirality— often overlooked— as a powerful control parameter for engineering active systems. The coupling of rotational motion, propulsion, and geometry opens up new strategies for directed assembly, pattern formation, and mechanical actuation in systems with active chiral constituents, such as biofilms, synthetic active crystals, or micromachines. Looking ahead, curvature‐driven mechanisms uncovered here may guide future studies on complex geometries, heterogeneous active mixtures, and torque‐controlled collective dynamics in microrobotics or soft active materials.

## 7 Experimental Section

### Simulation Method

Each active composite was composed of eight contacting spherical beads with diameter σ. The rodlike composites had a length of 8 σ and the circular ones had a radius of ≈ 1.3 σ. The unequal covered area by the two types of composites required that a different numbers of them be used to achieve a given occupied area fraction. The size of the simulation box is *L*  = 100 σ. The Lennard‐Jones potential in Equation (1) between interacting particles with center‐to‐center distance *d* is defined as

(3) $$U_{\text{LJ}}\left(d\right)=4\varepsilon \;\left[{\left(\frac{\sigma }{d}\right)}^{12}-\;{\left(\frac{\sigma }{d}\right)}^{6}\;\right],$$

where ϵ is the potential well depth. The mass of each bead *M*, its diameter σ, and the potential depth ϵ are chosen as the units of mass, length, and energy, respectively. The solvent friction is η  =  10 in all simulations. The Langevin dynamics simulations were carried using the LAMMPS code.^[^ ^58^, ^59^ ^]^ The integration time step was taken to be 10^−4^ τ, with $\tau \;=\;\sqrt{M\sigma^{2}/\varepsilon }$ being the simulation time unit. Before starting to measure the FI forces, A total of 10^7^ time steps was waited to reach the steady state in the presence of active forces. Next, the mean FI force acting on each intruder during ten time steps was measured. This quantity was averaged over 10^4^ successive time intervals. The process of measuring the time‐averaged forces were repeated over an ensemble of 10^2^ uncorrelated trajectories for a further reduction of the fluctuations.

## Author Contributions

J.S. and R.S. designed research; J.S., H.F., and H.K. designed and performed simulations; all authors contributed to analysis and interpretation of the results; R.S. wrote the paper; J.S. and R.S. revised the paper. Correspondence and requests for materials should be addressed to jalal@ipm.ir or shaebani@lusi.uni‐sb.de.

## Conflict of Interest

The authors declare no conflict of interest.

## Supporting information

Supplemental Movie 1

Supplemental Movie 2

Supplemental Movie 3

## Data Availability

The data that support the findings of this study are available from the corresponding author upon reasonable request.
